# Metastatic papillary thyroid carcinoma with internal jugular vein tumor thrombus - A case report and review of the literature

**DOI:** 10.3389/fendo.2025.1505800

**Published:** 2025-01-29

**Authors:** Zaina Adnan, Edmond Sabo, Sameer Kassem

**Affiliations:** ^1^ Department of Endocrinology and Metabolism, Clalit Medical Health Care Services, Haifa and Western Galilee District, Bar-Ilan Faculty of Medicine, Safed, Israel; ^2^ The Institute of Pathology, Carmel Medical Center, Ruth and Bruce Rappaport Faculty of Medicine, Technion-Institute of Technology, Haifa, Israel; ^3^ Department of Internal Medicine, Carmel Medical Center, Ruth and Bruce Rappaport Faculty of Medicine, Technion-Institute of Technology, Haifa, Israel

**Keywords:** case report, papillary thyroid carcinoma, tumor thrombus, distant metastasis, jugular vein, enhanced CT

## Abstract

Papillary thyroid carcinoma (PTC) is the most common malignancy of the thyroid gland, typically associated with an indolent course and favourable prognosis. However, although rare, PTC can demonstrate aggressive behaviour, including vascular invasion with extension into major vessels. Intraluminal tumor thrombus involving the great veins, such as the internal jugular vein (IJV), is an uncommon but significant complication. We present the case of a 56-year-old male who was referred to our clinic for evaluation of a right-sided anterior neck mass. Neck ultrasonography revealed a 5.5 x 6.5 cm heterogeneous mass within the right thyroid lobe and a suspected intraluminal thrombus in the right internal jugular vein. Fine-needle aspiration biopsy under ultrasound guidance confirmed the diagnosis of papillary thyroid carcinoma. Subsequent preoperative contrast-enhanced computed tomography (CT) of the neck confirmed the presence of an intraluminal tumours thrombus extending into the right IJV. The patient underwent total thyroidectomy, right modified radical neck dissection, and resection of the involved segment of the IJV. Postoperatively, the patient received radioactive iodine (I-131) ablation therapy. At the one-year follow-up, imaging studies indicated a recurrence of the disease. A review of the literature focusing on vascular involvement in PTC and diagnostic methods for tumours thrombus reveals that, while rare, intraluminal tumor thrombus should be considered in patients with PTC, especially when there is evidence of vascular invasion. Early and accurate preoperative diagnosis using Doppler ultrasonography and/or contrast-enhanced CT is critical for optimal surgical planning and improved prognosis. Given the potential for recurrence, vigilant long-term follow-up is recommended.

## Introduction

Papillary thyroid carcinoma (PTC)is common and accounts for approximately 80% of all thyroid malignancies (1). It is well known that some thyroid carcinoma may behave aggressively and tend to cause local invasion, recurrence, and distant metastasis (2). However, microscopic vascular invasion is common in all types of thyroid carcinoma (3). PTC rarely causes extension and growth that may involve the great veins, particularly the internal jugular vein, resulting in intraluminal tumor thrombus (4). In the current article, we present a rare case of metastatic PTC with intraluminal tumor thrombus in the internal jugular vein. We reviewed published data focusing mainly on vascular sites involved with tumor thrombus, diagnosis modalities, and the time of diagnosis preoperative vs. postoperative.

## Case presentation

**Figure 1:**
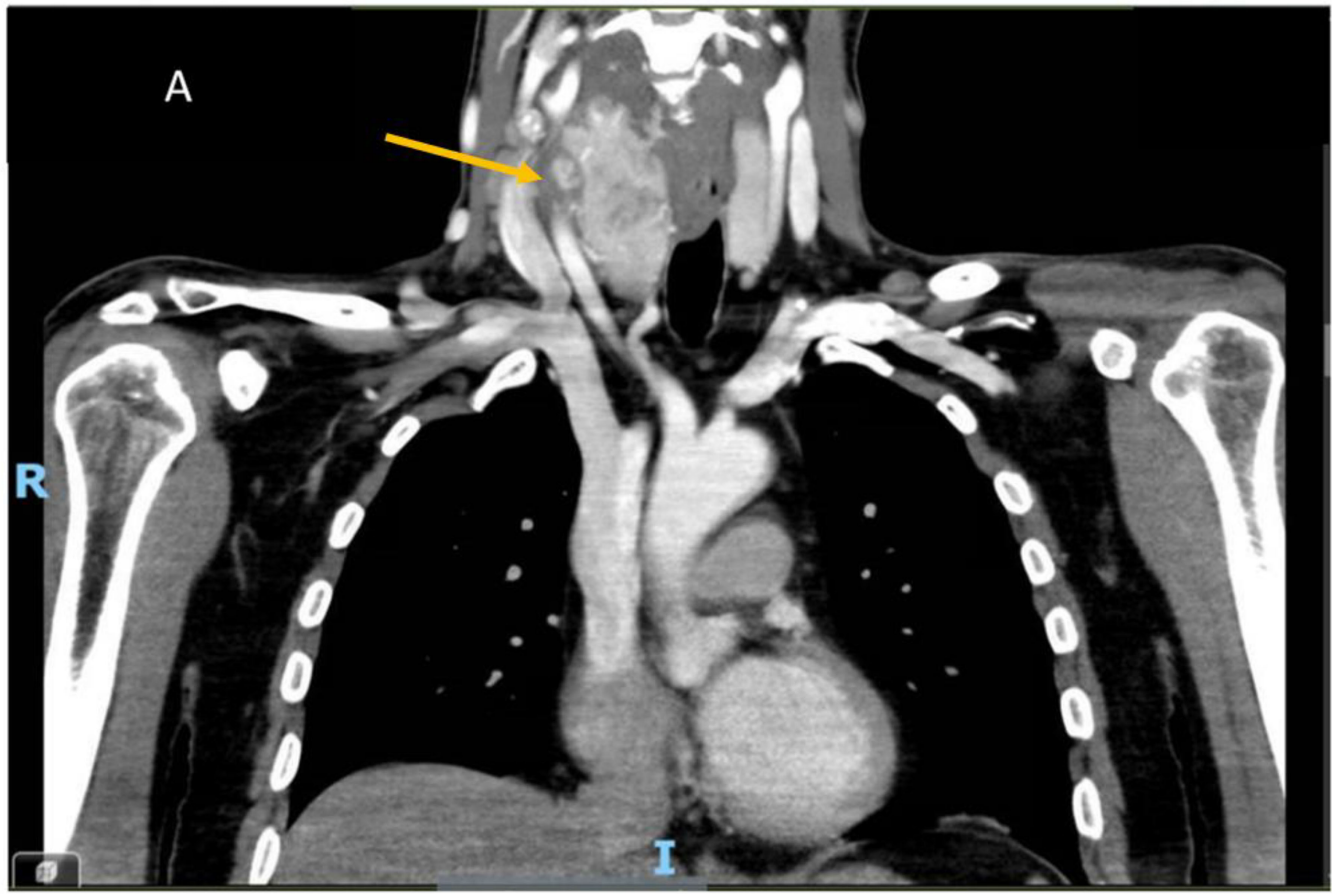
Contrast-enhanced computed tomography (CT) images of the neck and chest reveal a large nodule with areas of cystic degeneration within the right lobe of the thyroid gland. The yellow arrow indicates a tumor thrombus obstructing the right internal jugular vein (IJV).

A 56-year-old male was referred to our outpatient endocrinology department in July 2023 with a three-month history of progressive enlargement of a right-sided anterior neck mass. His past medical and family history were unremarkable. Physical examination revealed a firm, non-tender 5 x 5 cm mass within an enlarged right thyroid lobe without palpable lymphadenopathy. Neck ultrasonography identified a 5.5 x 6.5 cm heterogeneous mass in the lateral aspect of the right thyroid lobe, along with a suspected thrombus in the internal jugular vein (IJV). Fine-needle aspiration (FNA) guided by ultrasonography confirmed a diagnosis of papillary thyroid carcinoma (PTC). Preoperative enhanced contrast computed tomography (CT) of the neck and chest demonstrated a heterogeneous 5.5 x 6.5 cm right thyroid mass with hypodense areas and poorly defined margins at the base of the right neck, showing lateral extension. The right internal jugular vein (RIJ) was enlarged and heterogeneous, with an extensive filling defect consistent with an intraluminal tumor thrombus. Additionally, an 8 mm lymph node in the right upper lobe of the lung was noted, raising suspicion for metastasis, though there was no evidence of pericardial or pleural effusion (**Figure 1**).

**Figure 2:**
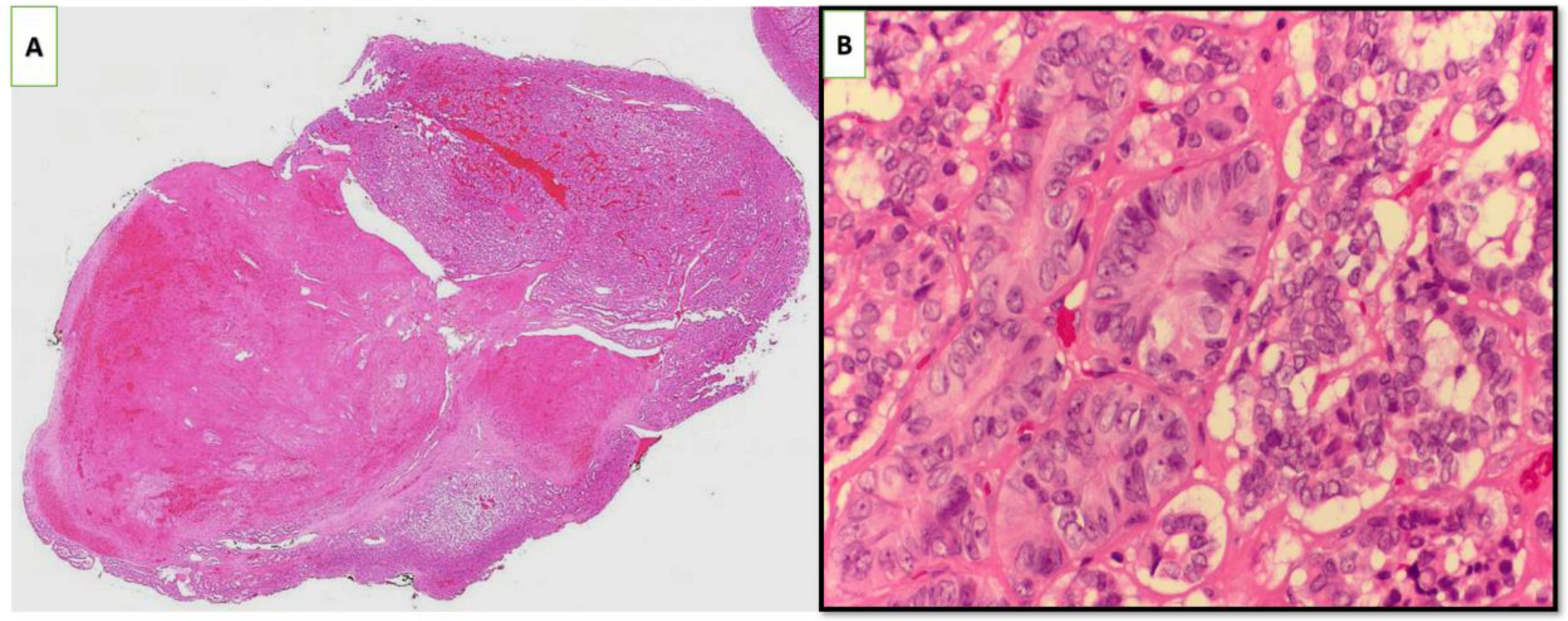
Histopathological examination revealed papillary thyroid carcinoma with a predominantly follicular growth pattern, along with areas consistent with poorly differentiated carcinoma. Vascular invasion was observed in a large vessel identified as the internal jugular vein, which contained a tumor thrombus (H&E staining; **(A)** ×10 magnification, **(B)** ×60 magnification).

After an extensive preoperative evaluation, including multidisciplinary consultation with a vascular surgeon team, in October 2023, the patient underwent a total thyroidectomy, right modified lymph node dissection, and resection of the internal jugular vein (IJV). Histopathological examination revealed a multifocal papillary thyroid carcinoma (PTC) measuring up to 7 cm, predominantly displaying a follicular growth pattern. There were cribriform and solid growth areas, with foci consistent with poorly differentiated carcinoma, constituting approximately 10-15% of the tumor volume. Evidence of vascular invasion was observed, including a large vein identified as the IJV containing a tumor thrombus. Invasion of the striated muscle was also noted. Metastatic carcinoma was detected in 3 of 26 lymph nodes, with the largest metastatic focus measuring 4 mm. The left thyroid lobe contained two foci of papillary thyroid microcarcinoma, each up to 5 mm, without extrathyroidal extension or vascular invasion. The IJV showed a tumor thrombus with fragments of PTC (**Figure 2**).

In February 2024, the patient underwent radioiodine ablation therapy with a dose of 150 mCi and was prescribed levothyroxine at 200 mcg daily. Laboratory results showed suppressed thyroid-stimulating hormone (TSH) levels and undetectable thyroglobulin (Tg) levels with normal thyroglobulin antibodies (TgAbs).

At the one-year follow-up in July 2024, a neck ultrasound revealed a suspicious right lymph node measuring 11 x 6.5 x 5 mm at level 3. Positron emission tomography/computed tomography (PET/CT) demonstrated pathological FDG uptake in a 10 mm lymph node in the right mid-neck, along with pulmonary nodules suggestive of lung metastasis. Fine-needle aspiration (FNA) indicated the lymph node was suspicious for metastatic PTC. In August 2024, the patient underwent resection of the lymph node and a left modified neck dissection. The histological findings revealed a 1.3 cm lymph node at level 3 on the right side, which was almost totally replaced by papillary metastatic carcinoma of a poorly differentiated variant. Focally extranodal tumor extension less than 1 mm was also seen. Examination of the excised lymph node at level 3 on the left neck disclosed fragments of lymph node tissue and surrounding fibro-fatty tissues infiltrated by metastatic poorly differentiated thyroid carcinoma variant. The other 13 dissected lymph nodes at this level were free of tumor involvement. At level 2, nine lymph nodes were dissected and found to be free of tumor. At level 4, one lymph node measuring 3 mm out of eight lymph nodes revealed metastatic thyroid carcinoma. no extranodal extension was observed. Postoperatively, a detectable Tg of 9.4 mcg/L with normal TgAbs was observed. Therefore, the patient was referred to the oncology department for further evaluation, ongoing follow-up, and treatment.

## Discussion

Tumour thrombosis of large vessels is commonly observed in angio-invasive malignancies, such as hepatocellular carcinoma and renal cell carcinoma, which frequently involve the portal vein, hepatic veins, renal veins, and the inferior vena cava (5, 6). In contrast, papillary thyroid carcinoma (PTC) typically spreads via the lymphatic system, with hematogenous dissemination leading to distant metastasis being rare (1). Microinvasion of cervical veins has been well documented in thyroid follicular and Hürthle cell carcinomas (2).

In this case report, we demonstrated the presence of an internal jugular vein (IJV) thrombus preoperatively. Reviewing the literature, we identified 50 cases of thyroid carcinoma associated with vascular tumor thrombus (**Table 1**). Among these patients, 34 were female, with a mean age of 56.8 years (range: 26–84 years). Most reports consist of case reports (34 cases) and case series (16 cases). Caudal extension of the thrombus was frequently observed, involving the brachiocephalic veins, superior vena cava, and, in some cases, propagation to the right atrium and tricuspid valve (7–44).

**Table 1:** Characteristics of patients with thyroid carcinoma and vascular tumor thrombus.

Author/year	n	Age/Sex	Pathology	Site of tumor thrombus	Diagnosis Pre/post operative	Diagnosis modality	Distant metastasis/ complication
Thompson et al. (7) 1978	1	67/F	FTC	IJV, BV, SVC, RA	preoperative	Angiography	Not reported
Thomas S et al. (8)1991	1	61/M	PDTC	IJV bilateral	Preoperative	Enhanced CT	Not reported
Onaran Yet al. (9) 1998	3	48/M	HCC	Lt IJV, BV, SV	Postoperative	Enhanced CT	Pituitary, T4-T5 vertebra
		48/F	PTC	IJV Rt	Preoperative	Doppler US	Not reported
		68/F	HCC	IJV Lt	Postoperative	Enhanced CT	Not reported
Wiseman O et al. (10) 2000	1	84/M	Thyroid ca.	Rt EJV, IJV, BV, SVC	Preoperative	Enhanced CT	Death before intervention
Koike E, et al. (11)2002	1	26/F	PDTC	BCV left	postoperative	Enhanced CT	Not reported
Yoshimura M et al. (5) 2002	1	65/F	ATC Giant cell	IJV SV Lt	preoperative	Gallium-67 scintigraphy	Not reported
Panzironi G. et al., 2003 (12)	1	68/F	ATC	IJV bilateral	preoperative Inoperapeble	Doppler US	Lung metastases.
Gross M et al (13) 2004	1	49/M	ATC HCC (Foci)	IJV Rt	Preoperative	Doppler US	Not reported
Ingle SA et al. (14) 2004	1	–	PTC	LT IJV Azygous vein	–	–	Superior VCS
Sugimoto S, et al. (15) 2006	1	61/M	Spindle cell ATC	Lt. IJV, BV bilat, SVC, RA TR	Preoperative	Enhanced CT MRI, I^131^ scintigraphy Venography	Lung metastasis
Taib NA et al. (16) 2007	3	66/F	FTC	Rt IJV SV, RA	postoperative	Enhanced CT	Not reported
		62/F	FTC	Rt IJV, SV, RA	Preoperative	Enhanced CT	Not reported
		45/F	FTC	IJV rt BV	preoperative	Enhance CT	Lung metastasis
Yamagami Y, et al. (17) 2008	1	74/M	PDTC	Lt IJV, BV SVC RA	Preoperative	Enhanced CT TEE	Not reported
Tripathi M, et al. (18) 2008	1	48/F	FTC Metastatic	Rt IJV BCV SVC	Postoperative	FDG-PET/CT CTA	Not reported
Hyer SL et al. (19) 2008	1	81/F	FTC	IJV Rt BV, SVC	Preoperative	Enhanced CT	Skull bone, Submandibular gland rt.
Agrawal A et al. (20) 2009	1	48/M	FVPTC	Rt IJV BV, Rt SV, SVC	Postoperative	I 131 WBS Enhanced CT	Not reported
Fotis T, et.al (21) 2009	1	49/F	PTC	Rt IJV LT IJV	Intraoperative	–	Superior VCS
Wada N, et al. (22) 2009	1	64/M	FTC	Lt IJV, BV, SVC	Preoperative	Enhanced CT	Not reported
Mugunthan N et al. (23), 2010	1	51/F	PTC	Lt IJV Lt BCV SVC, RA	Postoperative	Enhanced CT 131iodine	Not reported
Kobayashi K, et al. (24) 2011	6	75/F	PTC	Mid TV	Preoperative	Doppler US	Lung metastasis
		26/F	FTC	Lt IJV	Preoperative	Doppler US	Not reported
		69/F	PTC	Rt IJV	preoperative	Doppler US	Not reported
		77/M	PTC	Rt IJV	preoperative	Doppler US	Not reported
		80/F	Poorly DTC	Lt IJV	Preoperative	Doppler US	Lung metastasis
		35/F	PTC	Rt mid TV	intraoperative		Lung metastasis
Nakashima T et al. (25) 2012	1	54/M	FTC Poorly diff.	IJV bilat, TVs Lt BV, SV	Preoperative	Enhanced CT	Not reported
Babu S et al. (26) 2012	1	68/F	PTC	IJV Lt Lt BCV	Preoperative	Enhanced CT	Not reported
Onoda N, et al. (27) 2012	1	70/F	FTC	Rt IJV Rt BV SV, SVC	Preoperative	Enhanced CT	lung metastasis
Patten DK, et al. (28) 2012	1	54/M	MTC Metastatic	IJV	Preoperative	Doppler US	Not reported
Al-Jarrah Q. et al. (29) 2014	1	62/F	ATC/ PTC (thrombus)	IJV RT	Intraoperative	–	Not reported
Jafaripozve N, et al. (30) 2014	1	75/F	PTC	Rt IJV Superior TV	preoperative	Enhanced CT	Pulmonary embolism
Do Nascimento BB et al. (31) 2014	1	54/F	FTC	Lt IJV	preoperative	131 I-WBS Doppler US MRI	Not reported
Dikici et al. (32) 2015	1	52/F	PTC	LT IJV	Postoperative	–	Not reported
Franco IF et al. (33)2015	1	59/F	FTC	LT BV	Preoperative	Enhanced CT	Not reported
Manik G et al. (34) 2016	1	65/F	FTC	SVC, RA	Preoperative	Enhanced CT TEE	Not reported
Kawano F et al. (35) 2016	1	75/F	ATC	Lt IJV, BV, SVC, Lt sigmoid sinus	preoperative	Doppler US Thyroid scan FDG-PET Enhanced CT	Pulmonary embolism
Chiofalo MG et al. (36) 2018	3	75/F	FTC	Lt IJV BV	preoperative	Doppler US	Lung metastasis
		58/M	FTC	Rt IJV	preoperative	Doppler US	Lung and kidney metastasis
		64/F	FTC	Lt IJV	preoperative	Doppler US	Bone, lung metastasis
Botwe BO et al. (37) 2022	1	68/F	FTC	Lt IJV	Postoperative	–	Not reported
Ivanišević P et al. (38) 2020	1	67/M	FTC	Lt IJV, BV	Postoperative	–	Not reported
Rampelly S. et al. (39) 2020	1	50/M	PTC	LT IJV	Preoperative	Doppler US Enhanced CT	Not reported
Sezer H.et al (40) 2021	1	63/M	PTC Poorly diff.	Lt IJV	postoperative	MRI Neck	Not reported
Arun P, et al. (41) 2019	1	44 F	PTC	Lt IJV	Postoperative		Not reported
Lanks CW, et al. (42) 2023	1	53/F	PTC	Lt IJV	Preoperative		Pulmonary septic emboli Cerebellar stroke rt.
Yao J. et al. (43) 2023	1	62/F	PTC, Oncocytic (Collision)	Lt IJV SV	Preoperative	Doppler US Enhanced CT	Lung metastasis pneumothorax
Morvan JB, et al. (44) 2022	1	58/F	FTC Poorly Differentiated	Rt IJV Rt TVs	Preoperative	PET-CT Doppler US	Bone metastasis

PTC, Papillary thyroid carcinoma; FTC, Follicular thyroid carcinoma; HCC, Hürthle cell carcinoma; PDTC, Poorly differentiated thyroid carcinoma; ATC, Anaplastic thyroid carcinoma; FVPTC, Follicular variant of papillary thyroid carcinoma; TV, thyroid vein; IJV, Internal jugular vein; EJV, External jugular vein; SVC, Superior vena cava; SV, Subclavian vein; RA, Right atrium; BV, Brachiocephalic vein.

In contrast, proximal extension was rare, with only one case reporting involvement of the sigmoid sinus. Additionally, distant metastases to uncommon sites, including the vertebrae, pituitary gland, and skull bones, were reported (**Table 1**). Complications such as superior vena cava syndrome, pulmonary embolism, metastasis to different sites, and mortality were extensively reported in our manuscript.

Diagnosing vascular tumor thrombus can be clinically challenging and is highly dependent on the location and extent of the thrombus. However, dilated neck veins, upper limb edema, or extensive involvement of large vessels such as the jugular vein, subclavian vein, and superior vena cava should raise suspicion of tumor thrombus in this patient population. As highlighted in our case, preoperative imaging modalities, including Doppler ultrasonography (US) and contrast-enhanced computed tomography (CT), are essential for accurate diagnosis and surgical planning. Doppler US and contrast-enhanced CT were the most commonly used techniques to detect vascular tumor thrombus in the reviewed cases (**Table 1**). Contrast-enhanced CT is generally preferred due to its reliability, whereas the US is more operator-dependent, potentially limiting its accuracy.

## Conclusions

Tumor thrombus is rare among patients with PTC. However, this entity should be considered, particularly in patients with signs of vascular involvement. Preoperative diagnosis using Doppler US and/or enhanced contrast CT is mandatory for a favorable outcome.
